# The development of the PARENTS: a tool for parents to assess residents’ non-technical skills in pediatric emergency departments

**DOI:** 10.1186/s12909-017-1042-9

**Published:** 2017-11-14

**Authors:** Katherine A. Moreau, Kaylee Eady, Kenneth Tang, Mona Jabbour, Jason R. Frank, Meaghan Campbell, Stanley J. Hamstra

**Affiliations:** 1 Faculty of Education, University of Ottawa, 145 Jean-Jacques-Lussier Private, Ottawa, ON K1N 6N5 Canada; 2 Children’s Hospital of Eastern Ontario Research Institute, University of Ottawa, 401 Smyth Road, Ottawa, ON K1H 8L1 Canada; 3 School of Rehabilitation Sciences, Faculty of Health Sciences, University of Ottawa, 451 Smyth Road, Ottawa, ON K1H 8M5 Canada; 4 Department of Emergency Medicine, Faculty of Medicine, University of Ottawa, 1053 Carling Avenue, Ottawa, ON K1Y 4E9 Canada; 5 Department of Pediatrics, Faculty of Medicine, University of Ottawa, 401 Smyth Road, Ottawa, ON K1H 8L1 Canada; 6 Children’s Hospital of Eastern Ontario, University of Ottawa, 401 Smyth Road, Ottawa, ON K1H 8L1 Canada; 7 Royal College of Physicians and Surgeons of Canada, 774 Echo Drive, Ottawa, ON K1S 5N8 Canada; 8 Accreditation Council for Graduate Medical Education, 401 North Michigan Avenue, Chicago, IL 60611 USA

**Keywords:** Assessment, Validity, Parents, Pediatrics, Non-technical skills, Emergency

## Abstract

**Background:**

Parents can assess residents’ non-technical skills (NTS) in pediatric emergency departments (EDs). There are no assessment tools, with validity evidence, for parental use in pediatric EDs. The purpose of this study was to develop the **P**arents’ **A**ssessment of **R**esidents **E**nacting **N**on-**T**echnical **S**kills (PARENTS) educational assessment tool and collect three sources of validity evidence (i.e., content, response process, internal structure) for it.

**Methods:**

We established content evidence for the PARENTS through interviews with physician-educators and residents, focus groups with parents, a literature review, and a modified nominal group technique with experts. We collected response process evidence through cognitive interviews with parents. To examine the internal structure evidence, we administered the PARENTS and performed exploratory factor analysis.

**Results:**

Initially, a 20-item PARENTS was developed. Cognitive interviews led to the removal of one closed-ended item, the addition of resident photographs, and wording/formatting changes. Thirty-seven residents and 434 parents participated in the administration of the resulting 19-item PARENTS. Following factor analysis, a one-factor model prevailed.

**Conclusions:**

The study presents initial validity evidence for the PARENTS. It also highlights strategies for potentially: (a) involving parents in the assessment of residents, (b) improving the assessment of NTS in pediatric EDs, and (c) capturing parents’ perspectives to improve the preparation of future physicians.

**Electronic supplementary material:**

The online version of this article (10.1186/s12909-017-1042-9) contains supplementary material, which is available to authorized users.

## Background

Pediatric patients and their parents, guardians, or caregivers (herein referred to as parents) can be instrumental members of educational teams. One practical way for parents to engage in medical education is through the assessment of residents’ non-technical skills (NTS). Within medical education, the construct of NTS encompasses cognitive, social, and personal skills that work together to contribute to safe, efficient, and high quality health care [1]. These skills are the foundation of effective physician-patient/parent interactions. They are embodied within selected interwoven Roles (i.e., Communicator, Collaborator, Leader, Health Advocate, Scholar, Professional) of the CanMEDS physician competency framework developed by the Royal College of Physicians and Surgeons of Canada (RCPSC) [2]. This framework describes skills and abilities that residents must develop and demonstrate prior to certification for unsupervised clinical practice.

Pediatric Emergency Departments (EDs) provide excellent training environments for residents to develop and enhance their NTS. Residents must swiftly build positive relationships with patients and parents, provide quality care, and ensure that patients and parents understand the management of medical conditions. However, despite their importance, residents’ NTS are not well-assessed during ED rotations. Time and limited resources are cited as major barriers to the assessment of these skills [3, 4]. In busy EDs, it is challenging for supervising physicians to personally observe and assess all residents [5]. Yet, parents have personal experience of how residents interact with them and their children and can contribute to assessment processes [6–9]. Unfortunately, there are no assessment tools designed specifically for parents to assess residents’ NTS in pediatric EDs [10].

When considering patients’ perspectives, the medical education community has focused mainly on adult patients’ assessments of staff physicians. Boon and Stewart’s [11] systematic review merely reported one tool that involved parents of pediatric patients in physician assessment, by Street [12], with acceptable validity evidence. Likewise, Chisholm and Askham’s [13] review found only one tool for pediatric use. It is entitled the Sheffield Patient Assessment Tool (SHEFFPAT) [8] and has undergone sufficient validity and reliability testing. McGraw et al. [14] adapted this tool calling it the Paediatric Carers of Children Feedback tool (PaedCCF). McGraw and associates [14] have examined the reliability, validity, feasibility, and acceptability of using the PaedCCF in various pediatric clinics and conveyed positive findings. However, these above-mentioned tools and studies focus exclusively on staff physicians’ communication skills and outpatient clinic consultations, which often involve different care processes than EDs. As such, they are not designed for or completely relevant to residents in pediatric EDs. Moreover, these studies do not present multiple sources of validity evidence for the tools or use the validity framework of The Standards for Educational and Psychological Testing (The Standards) [15], which is considered best practice in the fields of psychometrics and medical education [16, 17]. Thus, there is a need to develop a new resident-focused educational assessment tool, with validity evidence, for parental use in pediatric EDs.

Informed by the validity framework of The Standards [15], the objective of the present three-phase sequential study was to develop the **P**arents’ **A**ssessment of **R**esidents **E**nacting **N**on-**T**echnical **S**kills (PARENTS) educational assessment tool and collect three sources of validity evidence for it: content, response process, and internal structure. The PARENTS is designed for parents of children 13 years of age or younger to formatively assess the NTS of residents who are their main health care providers during visits to pediatric EDs. The ultimate intention is that residents and physician-educators can use assessment scores and feedback from the PARENTS to help residents improve their NTS when interacting with pediatric patients and their parents.

## Methods

We conducted the study at a Canadian pediatric academic health science center and obtained ethics approval before data collection.

### Phase 1: Content evidence

To establish content evidence for the PARENTS we used four strategies. First, our study team, which included one parent, one resident, two physician-educators, three medical education researchers, and one statistician, met to clarify the formative purpose of the PARENTS and define the construct of NTS that is the focus of the tool. Second, to determine the format and generate items for the PARENTS, two members of the team facilitated dyadic interviews with physician-educators and residents (where two participants interacted with each other in one interview and responded to open-ended questions) as well as focus groups with parents who had visited a pediatric ED at least twice in the past year. At the beginning of these interviews and focus groups, we explained the intended purpose of our eventual PARENTS and provided the participants with background information on the construct of NTS, as embodied within selected interwoven Roles of the CanMEDS physician competency framework [2], which informed our conceptual basis for the creation of the educational assessment items. The participants explored which resident NTS parents in pediatric EDs can assess as well as the ideal format of the PARENTS. The interviews and focus groups lasted 30–60 min and were audiotaped and transcribed verbatim. As reported in Moreau et al. [18], the two study team members analysed the transcripts using three concurrent activities namely, data reduction, data analysis, and conclusions/verifications [19]. Based on the thematic findings, we formulated items for the draft PARENTS and placed the closed-ended items, depending on their wording, against a 4-point scale (where 1 = poor and 4 = outstanding, with an additional “Not applicable or not observed” option) or a “yes, no, I don’t know” scale.

Third, to add to these items, we conducted a literature search to identify items included on other instruments designed to assess patients’ and family members’ perceptions of medical students’, residents’, or staff physicians’ NTS. With the assistance of a librarian, we conducted a search in MEDLINE (see Additional file 1) and supplemented it with a PubMed related article search based on known relevant reports. One team member and a research assistant (RA) independently reviewed, extracted, and adapted items from the reviewed literature. We then compared their findings and resolved discrepancies through discussion.

Finally, since the 62-item PARENTS created through the above-mentioned processes was too long to pilot in a pediatric ED, as it would take parents significant time to complete, delay patient discharge or transfer, and negatively affect the provision of care, we invited physician-educators, residents, and parents, none of whom participated in the above-mentioned interviews or focus groups, to participate in a modified nominal group technique (NGT) [20]. To be eligible, physician-educators needed to self-identify as being involved in the assessment of residents in a pediatric ED for a minimum of 5 years, whereas residents had to have a minimum of 1 year of experience working in a pediatric ED. Parents needed to have visited a pediatric ED at least twice in the past year.

The NGT equally weighted the votes of each member [21].We provided each participant with the 62-item PARENTS, explained its purpose, and reviewed the construct of NTS. We then asked them the following question: What items are essential to include on the PARENTS? Each participant independently reviewed the 62 items and identified those that they wanted to include in the subsequent iteration. Next, using a round robin approach, each participant read aloud the item numbers that they wanted included, without naming any items others had previously mentioned, until all items were exhausted. We recorded the identified items, and had a brief discussion about the members’ rationales for wanting to include them. Each participant then voted confidentially and electronically on whether or not the various items should be included. Our goal was to reach a consensus level of 85% for each item [20]. If this level was not reached for selected items, the participants discussed them and re-voted. We did not limit the number of re-votes.

### Phase 2: Response process evidence

To collect response process evidence for the PARENTS, we used a cognitive interviewing technique to explore the ways in which parents understood, mentally processed, and responded to the items on the revised tool [22]. To increase assessment accuracy, we also solicited their opinions on how to administer the PARENTS. Recognizing that it was not feasible for parents to participate in a 1-h cognitive interview in a pediatric ED, we invited English-speaking parents who had a child 13 years of age or younger who was treated by a resident in the ED and transferred to an inpatient unit to participate. To gain insight from those with different experiences and with children of varying ages, we recruited parents from two general inpatient clinical teaching units that provide care to a wide-variety of patients and receive the highest number of patients from the ED. We used a cognitive interview protocol that included the revised PARENTS along with scripted probes. The probes focused on soliciting participants’ understandings of items, abilities to assess the NTS of the residents who cared for their children, interpretations of key concepts, wording suggestions, and level of difficulty associated with comprehending items. We also collected demographic information. The RA had each parent read each item on the tool and recorded the parent’s response to the item, which was based on his/her perception of the resident who treated his/her child in the ED. The RA then used concurrent probing to solicit feedback on each item. The RA took field notes during the interview and summarized the parent’s comments item-by-item.

Two study team members, with the RA, analyzed the field notes and item summaries by hand. They employed a pre-developed coding structure, informed by Willis, Schecter, and Whitaker’s [23] coding system. They classified data as a problem or solution. They coded data identified as a problem as: (a) a comprehension/communication problem (e.g., parents did not understand specific terms/words; parents struggled to assess a specific item); (b) a bias/sensitivity problem (e.g., parents thought that the item made it sound like they should answer it in a specific way); (c) a response option problem (e.g., the response options did not reflect the parents’ perceptions of the residents’ NTS); (d) logistic problem (e.g., parents’ perceptions of the item ordering or length of the items/tool); or (e) other problems (e.g., open coding category). They used inductive coding for the data classified as solutions. Given the number and nature of the problems/solutions, we determined that subsequent interview rounds were unnecessary and used the findings to make informed revisions to the PARENTS.

### Phase 3: Internal structure evidence

To examine the internal structure and to test the dimensionality of the PARENTS, we administered it over four 4-week resident training blocks in the ED. We invited all residents who attended the mandatory orientation session for their ED training block to participate. We invited parents who: (a) were English speaking, (b) had a child 13 years of age or younger who was receiving treatment in the ED by a participating resident, and (c) recognized the picture of the resident as his/her child’s ED health care provider. We excluded parents of children who required resuscitation. Based on the length of the PARENTS, we determined that we needed 340 completed assessments to attain a generous subject-to-item ratio of 20:1 for achieving a stable factor analysis solution [24].

To ensure that parents assessed their children’s residents, the RA photographed each consenting resident. To enroll parents, volunteer research assistants (VRAs) monitored the electronic patient tracking board from 10 am to 10 pm, 7 days/week to identify when a participating resident signed up as the health care provider for an eligible patient/parent (Note: Residents self-select their patients). A VRA then approached the parent immediately after his/her initial encounter with the participating resident, screened for eligibility, and provided a study package to be completed when the resident was not in the room. Residents did not know when parents were asked to assess them. All completed assessments were anonymous to minimize social desirability bias. Each package included the PARENTS and a demographic questionnaire. If more than one parent was present for an individual patient, the VRA invited only one parent to participate in order to maintain the independence of data. The parent then returned the completed package to a designated area prior to leaving.

We summarized participant demographic characteristics using descriptive statistics in SPSS version 24 (IBM, New York). To examine the underlying factor structure of the draft PARENTS, we conducted an exploratory factor analysis (EFA) with oblique (Geomin) rotation using M*plus* version 6.0 (Muthén & Muthén, Los Angeles, CA). We anticipated that the PARENTS would be unidimensional because the construct of NTS, as reflected within selected interwoven Roles of the CanMEDS physician competency framework [2], informed the creation of its items. However, based our study team’s review of the items generated in Phase 1 (Content Evidence), we decided to test three factor solutions in case the items potentially represented specific NTS grouping under the following CanMEDS Roles: Communicator, Collaborator, and Professional. Accordingly, we examined and compared 1-, 2-, and 3-factor solutions with the aim of identifying an optimal solution that is psychometrically sound and conceptually sensible. To inform psychometric performance, we examined and compared the eigenvalues (i.e. Kaiser’s criteria), model fit statistics (i.e. Root Mean Square Error of Approximation [RMSEA], Standardized Root Mean Square Residual [SRMR]), patterns of factor loadings (magnitude and indications of cross loadings), and factor correlations across the various factor solutions. We also computed Cronbach’s alphas for the items associated with each factor to determine internal consistency. We considered a coefficient of 0.70 or higher as acceptable.

In our EFAs, we applied corrections to the standard errors for non-independence of observations to account for the clustering of data. That is, since each resident cared for several patients in the ED, multiple observations from our dataset reflected parents’ ratings of the same resident (i.e. patients/parents were nested within residents). We also applied a robust weighted least squares estimator with mean and variance adjustment (WLSMV), which is considered the best option for modeling categorical or ordered data (i.e. does not assume normally distributed items) [25, 26] and is capable of handling varied response scales within the same tool. The WLSMV estimator handles missing data using the pairwise present method [26], which means each correlation is estimated using all available data for the given pair of items involved. We treated “I don’t know” and “Not applicable or Not observed” responses as missing values in the analysis.

## Results

### Phase 1: Content evidence

We interviewed six physician-educators and six residents. We also completed two focus groups with 22 parents (see Moreau et al. 2016 [18]). For the formatting of the PARENTS, we found that it should: (a) be in hardcopy format because of the unreliability of Wi-Fi and parents’ access to electronic devices in the ED; (b) include both closed- and open-ended items; (c) take approximately 5 min to complete; (d) protect the anonymity of the parent assessors and their children; (e) include a simple Likert-type scale for the closed-ended items; and (f) include a not applicable or not observed category in case some of the items do not apply to the parents or their children. As reported in Moreau et al. [18], we also found that parents can assess residents’ NTS, including their communication, comfort in a pediatric setting, adaptability, and collaboration. These findings resulted in the subsequent formulation of 27 items for the PARENTS.

Our literature search of other tools designed to assess patients’ and family members’ perceptions of medical students’, residents’, or staff physicians’ NTS identified 298 records. Following our screening, only 59 of these records discussed the composition of an assessment instrument. Our review of these 59 records identified 35 additional closed-ended items to include on the PARENTS, resulting in a 62-item PARENTS (see Additional file 2).

Ten parents, five residents, and five physician-educators participated in the modified NGT with the goal of decreasing the length of the PARENTS in order to feasibly pilot it in the ED. Through the voting process we developed a 20-item draft PARENTS, with 18 closed-ended and two open-ended items (see Table 1), which upon our study team’s review potentially related to NTS exemplified within the interwoven Communicator, Collaborator, and Professional Roles of the CanMEDS physician competency framework [2].

**Table 1:** Items retained in 20-item PARENTS

Closed-ended items from the focus groups and interviews
1. Did the resident introduce him/herself when meeting you and your child for the first time?
2. Did the resident identify him/herself as a resident?
3. How would you assess the resident’s skill to explain things in a way that you could understand?
4. How would you assess the resident’s skill to enter the room with some basic knowledge of your child’s condition?
5. How would you assess the resident’s skill to determine next steps about care or treatment with you, including any follow-up plans?
6. How would you assess the resident’s skill to listen to you and speak without interruption?
7. How would you assess the resident’s skill to understand what you had to say?
8. How would you assess the resident’s skill to interact with you comfortably?
9. How would you assess the resident’s skill to interact with your child comfortably?
10. How would you assess the resident’s skill to be flexible in his/her thinking and approach depending on your needs and those of your child?
Open-ended items from the focus groups and interviews
11. What can the resident do to improve his/her interactions with caregivers and their children?
12. Please use the space below to provide additional comments on the resident’s skills when interacting with you and your child?
Additional items from reviewed articles
13. Did the resident wash his/her hands?
14. Was the resident’s identification badge visible?
15. How would you assess the resident’s skill to pay full attention to you and your child during your interactions with him/her?
16. How would you assess the resident’s skill to discuss what to do if your child has any problems or complications related his/her condition?
17. How would you assess the resident’s skill to explain what he/she was doing for your child and why?
18. How would you assess the resident’s skill to explain your child’s treatment or prescribed medication, including possible side effects?
19. How would you assess the resident’s skill to show concern for your feelings and those of your child?
20. How would you assess the resident’s skill to answer your questions?

### Phase 2: Response process evidence

Twelve parents (6 mothers and 6 fathers) of children 13 years of age or younger treated in the pediatric ED by a resident and subsequently transferred to inpatient units participated in the cognitive interviews. All the parents were able to reflect on their experiences with residents in the ED. Each parent was able to provide examples to justify why he/she rated the resident the way that he/she did for each item. For example, one parent explained how the resident introduced himself as Dr. X but did not identify himself as a resident and therefore the parent answered “no” to the item “Did the resident identify him/herself as a resident?”. Whereas another parent described how the resident sat down to make eye contact with her and her child and felt that the resident was fully attentive to them and thus, answered “Very Good” to the item “pay attention to you and your child during your interactions with him/her”. Moreover, all parents were able to focus exclusively on the residents’ skills and did not overtly allow their interactions with other health professionals (e.g., nurses, staff physicians, social workers) or amount time spent in the ED waiting room influence their perceptions and ratings of the residents’ NTS.

The only significant interpretation difficulty that the parents had was with the meaning of the term *resident*. While each parent was able to recall the name of the resident who treated his/her child or provide an accurate physical description of the resident, all parents assigned various meanings to the term *resident*. As a way of reducing any ambiguity the parents thought it was important to define the term *resident* in the PARENTS’ written instructions. Additionally, to enhance the accuracy of assessments, parents advocated for the inclusion of a photograph of the resident who provided care to his/her child to ensure that the parent completed the assessment for the correct individual. The parents also suggested the removal of the item “Did the resident introduce him/herself when meeting you and your child for the first time?” because it was redundant to the item “Did the resident identify him/herself as a resident?”. Finally, the parents suggested changing the response option categories from “poor, marginal, good, outstanding” to “very poor, poor, fair, good, very good” as they better reflected their opinions/thoughts of the residents’ skills. They also recommended adding text to describe what each “very poor”, “poor”, “fair”, “good” and “very good” means (e.g., poor = the resident’s skill was inadequate. He/she needs a great deal of improvement; very good = the resident’s skill was truly noteworthy. He/she is a role model for others). Overall, the findings from the cognitive interviews led to minor formatting changes in the PARENTS, clearer instructions and response options, the inclusion of resident photographs, and ultimately the creation of a 19-item PARENTS (i.e., 17 closed-ended and two open-ended items).

### Phase 3: Internal structure evidence

Of the 46 residents who attended the orientation sessions, 37 (80%) participated in the study. Table 2 provides demographic information about the participating residents. During the study, 434 out of the 550 (79%) eligible parents approached participated. Table 2 also provides the parents’ demographic information.

**Table 2:** Demographic information for residents & parents

Characteristic	Number	n (%)
Residents		
Specialty	37	
Family Medicine		21 (57)
Pediatrics		6 (16)
Emergency Medicine		5 (14)
Psychiatry		3 (8)
Radiology		1 (3)
Dermatology		1 (3)
Postgraduate year	37	
PGY-1		28 (76)
PGY-2		4 (11)
PGY-3		3 (8)
PGY-4		1 (3)
PGY-5		1 (3)
Parents		
Patient age	429	
Less than 1 month		17 (4)
1–12 months		80 (18.8)
13–24 months		67 (15.7)
25–36 months		39 (9)
37–48 months		30 (7)
49–60 months		29 (6.7)
61–72 months		25 (5.8)
73–84 months		35 (8.2)
85–96 months		12 (2.8)
97–108 months		21 (4.9)
109–120 months		17 (3.9)
121–132 months		28 (6.5)
133–144 months		18 (4.2)
145–156 months		11 (2.6)
Relationship to the patient	432	
Father		125 (28.9)
Mother		292 (67.6)
Grandmother		4 (0.9)
Step-mother		1 (0.2)
Sister		2 (0.5)
Aunt		2 (0.5)
Parent		6 (1.4)

Participating parents reported high ratings of residents’ NTS. Table 3 provides the item-level descriptive statistics for the 17 closed-ended items of the PARENTS as well as the percentage of missing data for each item. The initial EFA on the 17 closed-ended items showed no factor loadings for items 1, 2, and 3. We therefore removed these items and ran a new EFA on the remaining 14 closed-ended items. We considered items to load onto a factor based on their strongest factor loading above 0.40. Table 4 presents the factor loadings for the 1-, 2-, and 3-factor models. The EFA showed that the 1-factor model is the most meaningful with a high eigenvalue of 10.93, which accounted for 78.1% of variance. Internal consistency for the single factor was high (α = 0.95). While the 2-factor model also appeared workable, its eigenvalue was below 1 (0.72), which accounted for 5.1% of variance. Factor 1 in the 2-factor model comprised items 5, 6, 8, 9, 10, 11, and 12 and factor 2 comprised items 4, 7, 13, 14, 15, 16, and 17. The internal consistency for factors 1 and 2 in the 2-factor model was high, α = 0.92 and 0.91, respectively. The 1- and 2-factor models correlated strongly together with a value of 0.86. The EFA also showed an eigenvalue below 1 for the 3-factor model with a value of 0.49. Moreover, it showed weak factor loadings but acceptable alpha values (α factor 1 = 0.92, factor 2 = 0.91, factor 3 = n/a [no items]). The 3-factor model correlated poorly with the 1- and 2- factor models, with a value of 0.32.

**Table 3:** Item and descriptive statistics for PARENTS (*N* = 434)

Item	Yes		No		N not applicable/not observed (%)	N non-response (%)
1: Did the resident identify him/herself as a resident?	350 (80.6)		19 (4.4)		15 (3.5)	50 (11.5)
2: Was the resident’s ID badge or nametag visible?	344 (79.3)		8 (1.8)		30 (6.9)	52 (12.0)
3: Did the resident wash his/her hands?	291 (67.0)		23 (5.3)		69 (15.9)	51 (11.8)
Item	Median	IQR	Mean	SD	N not applicable/not observed (%)	N non-response (%)
4: ...enter the room with some basic knowledge of your child’s condition?	5.00	1.00	4.43	0.75	8 (1.8)	5 (1.2)
5: ...listen to you and allow you to speak without interruption?	5.00	0.00	4.82	0.43	0 (0)	4 (0.9)
6: ...appear to understand what you had to say?	5.00	0.00	4.75	0.54	0 (0)	4 (0.9)
7: ...explain what he/she was doing for your child and why?	5.00	1.00	4.59	0.66	5 (1.2)	4 (0.9)
8: ...interact with you comfortably?	5.00	0.00	4.79	0.46	0 (0)	5 (1.2)
9: ...interact with your child comfortably?	5.00	0.00	4.73	0.53	2 (0.5)	4 (0.9)
10: ...be flexible in his/her thinking and approach depending on your needs and those of your child?	5.00	0.00	4.65	0.59	30 (6.9)	6 (1.4)
11: ...show concern for your feelings and those of your child?	5.00	0.00	4.69	0.57	9 (2.1)	7 (1.6)
12: ...pay attention to you and your child during your interactions with him/her?	5.00	0.00	4.74	0.56	3 (0.7)	9 (2.1)
13: ...explain your child’s treatment or prescribed medication, including possible side effects?	5.00	1.00	4.60	0.69	108 (24.9)	18 (4.1)
14: ...determine next steps about care or treatment with you, including any follow-up plans?	5.00	1.00	4.62	0.68	83 (19.1)	18 (4.1)
15: ...discuss what to do if your child has any problems or complications related to his/her condition?	5.00	1.00	4.60	0.71	116 (26.7)	19 (4.4)
16: ...answer your question?	5.00	0.00	4.72	0.57	37 (8.5)	13 (3.0)
17: ...explain things in a way that you could understand?	5.00	0.00	4.75	0.54	9 (2.1)	14 (3.2)

**Table 4:** EFA factor loadings for the 1-, 2-, and 3-factor models

	1-factor	2-factor		3-factor		
	1	1	2	1	2	3
Q4	**0.771**	0.283	**0.522**	0.230	**0.567**	0.030
Q5	**0.867**	**0.866**	0.021	**0.825**	−0.003	0.196
Q6	**0.875**	**0.747**	0.159	**0.685**	0.145	0.235
Q7	**0.851**	0.357	**0.533**	0.308	**0.654**	−0.203
Q8	**0.896**	**0.914**	0.005	**0.852**	0.083	−0.011
Q9	**0.876**	**1.047**	−0.169	**0.990**	−0.017	−0.230
Q10	**0.918**	**0.555**	0.404	**0.500**	0.450	0.043
Q11	**0.887**	**0.858**	0.058	**0.793**	0.131	0.011
Q12	**0.891**	**0.924**	−0.013	**0.830**	0.128	−0.101
Q13	**0.895**	0.070	**0.858**	0.078	**0.899**	−0.160
Q14	**0.920**	−0.015	**0.950**	−0.029	**1.043**	−0.218
Q15	**0.897**	−0.070	**0.977**	−0.085	**0.989**	0.003
Q16	**0.944**	0.083	**0.887**	0.006	**0.864**	0.259
Q17	**0.906**	0.171	**0.762**	0.148	**0.699**	0.221
Alpha	0.95	0.92	0.91	0.92	0.91	N/A
RMSEA	0.073	0.057		0.046		
SRMR	0.051	0.030		0.022		

Values in bold indicate the strongest factor loading for each item

*RMSEA* root mean square error of approximation, <0.08 = acceptable, <0.05 = ideal

*SRMR* standardized root mean square residual, <0.08 = acceptable, <0.05 = ideal

## Discussion

In this study, we sought to collect three sources of validity evidence for our novel PARENTS educational assessment tool, namely content, response process, and internal structure. In Phase 1, we successfully established the formative purpose of the PARENTS and generated possible items for it, which related to the construct of NTS embodied potentially within selected Roles of the CanMEDS physician competency framework [2]. We also solicited experts’ opinions on the crucial items to include on the PARENTS. By including physician-educators, residents, and parents in the development of the items, we were able to gather a range of perspectives, establish checks and balances for the participants, and thus, minimize the creation of items that would lead inevitably to extremely negative or exceedingly positive ratings. In Phase 2, we used cognitive interviews to explore the response process of parents as they reviewed and completed the PARENTS. Through these interviews we obtained evidence that parents are able to use the PARENTS to assess residents’ NTS. They also reported to exclude irrelevant factors from their judgments (e.g., interactions with other health professionals, wait times, and overall satisfaction with the ED care experience).

Furthermore, while the participants provided concrete examples of what the residents did or did not do to justify their ratings for the various items, their comprehension of the term *resident* is not surprising. Hemphill et al. [27] in a survey of adult patients and their families in an American ED found that while the respondents believed it was imperative to know and fully understand their physicians’ training level (e.g., medical student, resident, attending), the majority did not comprehend the roles and responsibilities expected of physicians at different training levels. As such, in a follow-up study, Santen et al. [28] recommended providing patients and their families with information on the levels of medical education and explaining the roles and responsibilities of the physicians in training who may be involved in their care. Given these important recommendations as well as those of the parents in Phase 2 of our study, we included a definition of the term *resident* at the onset of our assessment tool. To confirm that the parents assess the correct individual, the participants in Phase 2 also recommended showing parents a photograph of the resident who provided care to his/her child. This strategy was also introduced and used successfully by Brinkman et al. [5] to ensure that various assessors recognized and assessed the correct individuals within the inpatient setting for their study. Additionally, in Phase 2 the parents believed that the original 4-point response option scale failed to capture their perceptions of the residents’ skills. Their suggestion of converting the 4-point response option scale to a 5-point one is well supported by research on Likert-type rating scales, as it is balanced with an equal number of positive and negative options [29].

In regards to Phase 3, internal structure evidence, we found there were no factor loadings for items 1, 2, and 3 on the PARENTS. This suggests that these items are discrete and can, in future, be scored and reported individually. The EFA also showed that there is one plausible factor model for items 4 to 17 on the tool. This model has a high Cronbach’s alpha and shows that the included 14 closed-ended items group together well. This indicates the potential for summing items 4 to 17 to create one total score for each resident out of 70. From an educational and clinical perspective, this one factor model is not surprising because the items on the PARENTS all relate to and assess residents’ enactment of NTS. As mentioned, these NTS are reflected within selected interwoven Roles (i.e., Communicator, Collaborator, Professional) of the CanMEDS physician competency framework [2]. Thus, they are often taught, demonstrated, and assessed together in order to promote respectful and compassionate interactions between physicians and their patients/parents, to enhance family-centered care [30], and to provide safe, efficient, and high quality health care [1]. Based on this information, we can retain the PARENTS as a 19-item tool (i.e., 17 closed-ended items and 2 open-ended items).

A major strength of this study is that we engaged parents in it from the onset and co-created the research *with* them (rather than *on* them). We included parents in the design of the study, solicited their perspectives in the development of the PARENTS, and invited them to participate in dissemination. Through the ongoing development and future use of the tool, we hope to continue to build authentic partnerships with parents [31], gain parents’ insights into resident-patient-parent interactions, use parents’ assessment scores and feedback to improve these interactions [30], and exemplify that parents can be a valuable resource in the assessment and improvement of NTS.

The residents’ scores along with open-ended comments could be integrated into daily formative feedback that residents receive in the ED. We are optimistic that residents will use the assessment scores and feedback from the PARENTS for improving their NTS when interacting with pediatric patients and their parents. Given that the parents reported high ratings of residents’ NTS, residents could, for example, use the scores and feedback to build confidence in their NTS and confirm the strength of their NTS in pediatric patient/parent interactions [32, 33]. Conversely, physician-educators could use the scores and feedback to identify residents with atypical ratings (i.e., low scores, weak NTS) and offer educational remediation to help them improve their NTS when interacting with pediatric patients and their parents.

Another strength of this study is that we used the validity framework of The Standards [15]. In contrast to the classical conceptualization of validity, where validity is a possession of the instrument itself (e.g., “validated instrument”) and has distinct types (e.g., content, criterion, construct), this framework presents validity as a unitary concept and as the degree to which evidence supports the intended interpretation of assessment scores for a given purpose [15]. While the framework outlines five sources of validity evidence that should be collected (i.e., content, response process, internal structure, relations to other variables, consequences), literature reviews show that few researchers collect multiple sources of validity evidence for assessment instruments and that they commonly misinterpret and misapply the concept of validity [17, 34] thereby, potentially using poorly developed tools to inform educational and clinical practices. We collected three sources of validity evidence for the PARENTS. As a next step, we are also exploring the consequential evidence of the results and interpretations of the tool by investigating if and how residents and physician-educators use the scores and feedback from the PARENTS as well as the extent to which it influences their clinical practices.

This study also has some limitations, which future studies can mitigate. First, this study was conducted in one Canadian pediatric setting with a convenience sample of parents, residents, and physician-educators. We recognize the value of conducting additional psychometric studies to further evaluate the validity and reliability of the PARENTS’ scores and interpretations in other pediatric contexts. Such studies would also help us better map the assessment scores of the PARENTS to the broad underlying construct of NTS. Second, since this was the first administration of the PARENTS, it is not known if the tool is able to show changes in residents’ NTS over time. It would be interesting to follow a group of residents and have parents complete the tool at various time points to see if residents’ NTS improve as they acquire more clinical experience or after they complete educational interventions targeted at improving their NTS. This type of investigation would provide further evidence of validity (i.e., relations to other variables). Third, although the parents in this study reported that they excluded, for example, interactions with other health professionals, wait times, and global satisfaction with ED care from their assessments of the residents, future studies could investigate if there are correlations between residents’ scores on the PARENTS and these factors. Such studies would provide insights on the relationships (or lack thereof) that these factors have with parents’ assessments of residents’ NTS. Fourth, despite encouraging parents to answer all the items on the PARENTS, some of the 17 closed-ended items had moderate levels of missing data. Non-responses to items 1–3 were more frequently observed than other items because we suspect respondents had accidently bypassed the top portion of the PARENTS, where these questions were located (we have now subsequently revised the layout). In addition, a non-trivial proportion (>15%) of “not applicable/not observed” responses was also noted for items 3, 13, 14, and 15. Although we do not consider these proportions to be excessively high to the point of being concerning in terms of item relevance, it would be interesting to further investigate, for example, why parents did not observe or know if the resident washed his/her hands. Fifth, while we took steps to ensure that the items on the PARENTS were framed properly (e.g., non-leading, worded clearly) and that the 5-point response option scale was balanced and appropriate, in Phase 3, we still found that the parents’ assessments of the residents were positively skewed. The skewness of the data may have also contributed to a more simplistic factor solution (i.e., 1 factor model) being favored over more complex factor solutions (i.e., 2 or 3 factor models). In forthcoming studies, we will investigate potential causes of this skewness and determine if there are additional steps we can take to ensure that the PARENTS is able to capture variation in residents’ NTS. We will also examine if the scores on the PARENTS are a reflection of the residents’ strong NTS or a function of the tool itself namely, its inability to discriminate between residents. Specifically, in order to further explore the relations to other variables validity evidence of the PARENTS, we will compare residents’ PARENTS results to an established reference group known to have weaker NTS to determine if the tool can sort individuals accordingly. Sixth, in the present study we did not investigate if the PARENTS scores varied by participant characteristics. It would be valuable to create hypotheses and investigate if the PARENTS scores varied by: (a) severity of the patients’ condition(s), (b) patients’ age, (c) assessors’ relationship to the child, (d) residents’ specialties, or (e) residents’ training levels. Lastly, we do not fully know how parents perceived their experience of assessing residents. While we heard anecdotally that it had positive impacts on them, it is possible that not all parents reacted positively towards their involvement and thus, further investigation is needed in this area.

## Conclusions

This study describes initial yet important efforts to create a tool designed specifically for parents to assess residents’ NTS in pediatric EDs and establish validity evidence for it. It shows promising validity evidence for a 19-item (i.e., 17 closed-ended items and 2 open-ended items) PARENTS. It also highlights strategies for involving parents in resident assessment and potentially improving the assessment of NTS in pediatric EDs. Furthermore, it exemplifies that it is feasible to engage patients’ family members in medical education research. By actively involving these individuals, we can reassess our research priorities, gain new insight on key topics, and ultimately, ensure that patients and their families truly remain the focal point of our medical education endeavours.

## Additional files

Additional file 1: MEDLINE search strategy. Search strategy to identify items included on other instruments designed to assess patients’ and family members’ perceptions of medical students’, residents’, or staff physicians’ NTS. (DOCX 11 kb)

Additional file 2: Items included on the 62-item PARENTS. A list of items included on the 62-item PARENTS. (DOCX 15 kb)

## Abbreviations

ED: Emergency Department
EFA: exploratory factor analysis
NGT: nominal group technique
NTS: non-technical skills
PaedCCF: Paediatric Carers of Children Feedback tool
PARENTS: Parents’ Assessment of Residents Enacting Non-Technical Skills
RA: research assistant
RCPSC: Royal College of Physicians and Surgeons of Canada
SHEFFPAT: Sheffield Patient Assessment Tool
The Standards: The Standards for Educational and Psychological Testing
VRAs: volunteer research assistants
WLSMV: weighted least squares estimator with mean and variance adjustment
