# Supplementary material for: The development of the PARENTS: a tool for parents to assess residents’ non-technical skills in pediatric emergency departments
Source: BMC Med Educ. 2017 Nov 14;17:210. doi: 10.1186/s12909-017-1042-9 (PMC5686846; doi:10.1186/s12909-017-1042-9)
Supplement: Supplementary file 1 — MEDLINE search strategy. Search strategy to identify items included on other instruments designed to assess patients’ and family members’ perceptions of medical students’, residents’, or staff physicians’ NTS. (DOCX 11 kb) [file 12909_2017_1042_MOESM1_ESM.docx]

Additional File 1

*MEDLINE search strategy*

1. Clinical Competence/

2. (nontechnical skill* or non-technical skill*).tw.

3. Professional-Family Relations/

4. Physician-Patient Relations/

5. Patients/

6. Perception/

7. (1 or 2) and (or/3-6)

8. Reproducibility of Results/

9. Questionnaires/

10. 8 or 9

11. 7 and 10

12. Parents/ or pediatric/ or (child or adolescent or infan*).mp.

13. 11 and 12
