# Supplementary material for: The development of the PARENTS: a tool for parents to assess residents’ non-technical skills in pediatric emergency departments
Source: BMC Med Educ. 2017 Nov 14;17:210. doi: 10.1186/s12909-017-1042-9 (PMC5686846; doi:10.1186/s12909-017-1042-9)
Supplement: Supplementary file 2 — Items included on the 62-item PARENTS. A list of items included on the 62-item PARENTS. (DOCX 15 kb) [file 12909_2017_1042_MOESM2_ESM.docx]

Additional File 2

*Items included on the 62-item PARENTS*

| **Closed-ended items** |
| --- |
| 1. **Did the resident introduce him/herself when meeting you and your child for the first time?** |
| 1. How would you assess the resident’s ability to explain his/her role in the Emergency Department (ED)? |
| 1. **Did the resident identify him/herself as a resident?** |
| 1. **How would you assess the resident’s skill to explain things in a way that you could understand?** |
| 1. **How would you assess the resident’s skill to enter the room with some basic knowledge of your child’s condition?** |
| 1. How would you assess the resident’s skill to translate medical information to you and your child at discharge? |
| 1. **How would you assess the resident’s skill to determine next steps about care or treatment with you, including any follow-up plans?** |
| 1. Did the resident provide you with adequate follow-up instructions at discharge? |
| 1. How would you assess the resident’s skill to summarize the information you provided? |
| 1. **How would you assess the resident’s skill to listen to you and speak without interruption?** |
| 1. **How would you assess the resident’s skill to understand what you had to say?** |
| 1. How would you assess the resident’s ability to understand your reason(s) for coming to the ED? |
| 1. How would you assess the resident’s skill to keep you informed about your child’s care in the ED? |
| 1. How would you assess the resident’s skill to coordinate your child’s care in the ED? |
| 1. How would you assess the resident’s skill to explain how the ED operates (e.g., organizational processes)? |
| 1. **How would you assess the resident’s skill to interact with you comfortably?** |
| 1. **How would you assess the resident’s skill to interact with your child comfortably?** |
| 1. Was the resident confident when he/she was examining your child? |
| 1. **How would you assess the resident’s skill to be flexible in his/her thinking and approach depending on your needs and those of your child?** |
| 1. How would you assess the resident’s skill to collaborate with other health professionals in the ED? |
| 1. Did the resident trust the information you provided? |
| 1. Was the resident empathic? |
| 1. Did you feel included in your child’s care, as a participant in it, rather than just a bystander? |
| 1. Was the resident respectful of other members of the healthcare team? |
| 1. Did the resident contact the appropriate specialists for your child? |
| 1. Was the resident dressed appropriately? |
| 1. **Did the resident wash his/her hands?** |
| 1. **Was the resident’s identification badge visible?** |
| **Closed-ended items continued** |
| 1. How would you assess the resident’s ability to know his/her limitations (e.g., admit mistakes or lack of knowledge)? |
| 1. How would assess the resident’s skill to communicate with other health professionals in an efficient manner? |
| 1. **How would you assess the resident’s skill to pay full attention to you and your child during your interactions with him/her?** |
| 1. How would you assess the resident’s ability to maintain privacy and confidentiality? |
| 1. Did the resident provide you with reports and letters in a timely manner? |
| 1. Did the resident display cultural awareness? |
| 1. Did the resident display cultural sensitivity? |
| 1. How would you assess the resident’s skill to listen to the patient’s concerns? |
| 1. Did the resident encourage you to ask questions? |
| 1. Did the resident ensure that you understood what was going on? |
| 1. **How would you assess the resident’s skill to discuss what to do if your child has any problems or complications related his/her condition?** |
| 1. Did the resident use non-verbal communication effectively (e.g., smiled, made eye contact)? |
| 1. **How would you assess the resident’s skill to explain what he/she was doing for your child and why?** |
| 1. How would you assess the resident’s skill to clearly explain medical problems to you? |
| 1. How would you assess the resident’s skill to clearly explain treatment options to you? |
| 1. **How would you assess the resident’s skill to explain your child’s treatment or prescribed medication, including possible side effects?** |
| 1. **How would you assess the resident’s skill to show concern for your feelings and those of your child?** |
| 1. Did the resident respect your family’s beliefs/ideas/perspectives/opinions/preferences? |
| 1. Did the resident define family and physician roles in care? |
| 1. Did the resident share decision making responsibilities with you and the patient? |
| 1. How would you assess the resident’s skill to keep necessary health professionals informed and up to date about the patient? |
| 1. How would you assess the resident’s ability to build a treatment plan to fit your child? |
| 1. Did the resident spend an appropriate amount of time with you and your child? |
| 1. Was the resident available when you or your child needed him/her? |
| 1. How would you assess the resident’s ability to help you gain access to necessary resources (e.g., community resources, additional information about a condition, psychosocial support)? |
| 1. How would you assess the resident’s knowledge of your neighborhoods’ health concerns? |
| 1. How would you assess the resident’s ability to provide education to you on your child’s condition or problem? |
| 1. How would you assess the resident’s skill to handle difficult situations (e.g., serious illness, end of life)? |
| 1. How would you assess the resident’s ability to handle a difficult child? |
| 1. How would assess the resident’s ability to work effectively with other health professionals? |
| **Closed-ended items continued** |
| 1. How would you assess the resident’s skill to explain the rationale for tests and treatments? |
| 1. **How would you assess the resident’s skill to answer your questions?** |
| **Open-ended items** |
| 1. **What can the resident do to improve his/her interactions with caregivers and their children?** |
| 1. **Please use the space below to provide additional comments on the resident’s skills when interacting with you and your child?** |

*Note.* Bolded items retained in 20-item PARENTS
